# Supportive Care in Older Lymphoma Patients to Reduce Toxicity and Preserve Quality of Life

**DOI:** 10.3390/cancers15225381

**Published:** 2023-11-13

**Authors:** Fulvio Massaro, Fabio Andreozzi, Charlotte Vandevoorde, Dominique Bron

**Affiliations:** Department of Hematology, Jules Bordet Institute, Hôpital Universitaire de Bruxelles, Université Libre de Bruxelles, 1070 Brussels, Belgium; fulvio.massaro@hubruxelles.be (F.M.); fabio.andreozzi@hubruxelles.be (F.A.); charlotte.vandevoorde@hubruxelles.be (C.V.)

**Keywords:** lymphoma, supportive care, elderly, older patients, quality of life, geriatric assessment, frailty

## Abstract

**Simple Summary:**

Lymphoproliferative disorders are commonly observed in the elderly population and usually their treatment can cause numerous side effects, loss of autonomy and impaired quality of life. The management of these patients, when a curative treatment is proposed, needs to be personalized according to subject, disease and treatment features. Nowadays, in order to reduce the impact of toxicities, supportive therapy is a crucial ally during the administration of chemo-immunotherapy. Particularly, the prevention of hematological and infectious complications, tumor lysis syndrome and cardiovascular and neurological events could lead not only to improved oncological outcomes but also to improved quality of life.

**Abstract:**

The treatment paradigm in older patients with malignant hemopathies is the choice between an effective conservative treatment that preserves quality of life and an intensive, potentially curative treatment with more toxicities. For each patient, it is important to determine the risk/benefit ratio. The patient should be involved in the discussion, sufficiently informed and able to express himself and his expectations in terms of quality of life. However, this informed consent is conditioned by the ability of the patient to understand the risks and benefits of the treatment. Decline in quality of life is an important parameter for older patients with cancer and many prospective trials have now confirmed the impact of different side effects of treatment, such as recurrent hospitalization, loss of autonomy in daily activities, loss of contact with grandchildren and loss of cognitive functions. Interventions oriented to vulnerabilities detected in the older patients (by comprehensive geriatric assessment) and an optimal approach, including preventive measures to reduce treatment-related toxicity and mortality, are directly correlated to improvement in quality of life.

## 1. Population Aging: A Challenge for Oncologists

### 1.1. Malignant Hemopathies: Diseases of Older Patients

The median age of the population is increasing and, within 10 years, it is expected that 30% of the European population will be older than 60 years. This represents a challenge for hemato-oncologists who will have to face the increased incidence of malignant hemopathies with a median age above 65 years [1,2,3].

Lymphomas are the most common hematological malignancies, accounting for 30% of these malignant hemopathies. Diagnosis and treatment of lymphoma patients have become increasingly complex, with new approaches such as bispecific antibodies and CAR-T cells therapies showing specific side effects [4,5,6,7].

Our priority should be focused on identifying older patients who could benefit from specific treatment adapted to their “vulnerable” status and those who no longer benefit from classical therapy. Preserving patients from treatment-related toxicity and mortality or loss of autonomy is one of the keys to maintaining their quality of life (QoL) [8].

### 1.2. Older “Fit” Patients Are “Vulnerable”

Older patients require special consideration because comorbidities may compromise their tolerance to the “curative” treatment and lead to life-threatening side effects.

To investigate the fitness status of older patients, the optimal approach remains the “Comprehensive Geriatric Assessment” (CGA) [9,10]. A complete evaluation of older patients using different scales, investigated by an onco-nurse or geriatrician, will detect unsuspected vulnerabilities and will allow an adequate intervention in order to reduce chemotherapy-related side effects [11,12].

However, to optimize the treatment of older patients, beyond CGA, we should pay attention to further defining the nature of the problem, as:

1. Patient-related?
2. Disease-related?
3. Treatment-related?

**Figure 1:**
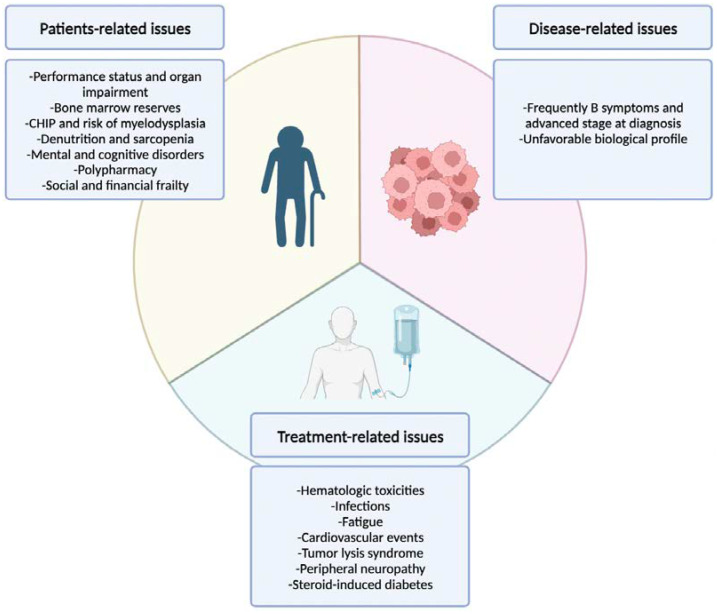
Summary of the medical issues related to elderly lymphoma patient management.

These features are summarized in Figure 1.

#### 1.2.1. Patient-Related Issues

Ageing leads naturally to the gradual damage of physiological functions, an increased inflammatory status, reduced naïve immune T-cell population, new mutational events and epigenetic changes resulting in a weakened health status [13]. This loss of physiological functions due to the normal ageing process constitutes the main cause of frailty but it is also affected by associated diseases.

It is clear today that chronological age alone is meaningless. This population is heterogeneous in terms of comorbidities, life expectancy, physical fitness and socio-economic situation [14]. Even in very “fit” patients, marrow reserves and renal function are reduced and neurological tolerance to toxic drugs is severely impaired. Clonal hematopoiesis of indeterminate potential (CHIP), which is associated with an increased risk of myelodysplasia, cardiovascular diseases and greater overall mortality, is another consequence of ageing [15,16]. In very old patients, we must also pay attention to geriatric syndromes (falling, cognitive disorders including dementia, incontinence and loss of autonomy) associated with a shorter life expectancy. 

In an exhaustive multivariate analysis from M. E. Hamaker, poor physical capacity, (low performance status or poor “up and go” evaluation), nutritional deficits and, finally, the comorbidity index are the three major predictive factors of mortality in older patients with hematologic malignancies [9]. Lodewijckx, in a Belgian survey, also confirmed the predictive factors for unacceptable toxicities such as loss of independence, early toxic death and unexpected hospitalization. The significant predictive factors were Mini Nutritional Assessment (MNA) score < 24, “up and go” test > 20 s, Instrumental Activities of Daily Living (IADL) score < 8 and a Geriatric Depression Scale-15 (GDS-15) > 5 [17]. 

Many frailty scores in various studies confirmed the impact of these common features: nutritional status, cognition and physical performance combined with inflammation status [18,19]. Taking into account four independent prognostic factors (low serum albumin, patient comorbidities (two or more), a Mini Mental State Evaluation (MMSE) < 27 and increased C-reactive protein), we also proposed a simple frailty score (HEMA-4) to be used even in “clinically fit” patients referred to receive chemotherapy for lymphoproliferative disorders. We observed that, whatever the disease or the status of the disease, the presence of these four parameters was associated with a median overall survival (OS) of less than 6 months, significantly different (*p* < 0.001) when compared to age-matched patients with none of these prognostic factors [20].

There is a general consensus that neither age nor ECOG score is sufficient to evaluate frailty and to enable physicians to propose a treatment adapted to “vulnerability” [21]. 

While physicians are quite efficient in evaluating functional status or comorbidities, other vulnerabilities such as nutritional status, polypharmacy and psycho-cognitive functions are usually poorly investigated and socio-economic status is rarely evaluated [10,18].

Therefore, to better evaluate the fitness of older patients, close collaboration between geriatricians and hematologists is highly recommended.

Comprehensive Geriatric Assessment (CGA) investigates all the aspects of the older patient (physiology, psychology, cognition, socio-economical status) and has been validated in a recent review [10]. Unsuspected vulnerabilities such as polypharmacy and malnutrition are detected, respectively, in 51% and 44 % of the patients. Cognitive impairment exists in 17% of the investigated patients. This assessment resulted in treatment modification in one third of the subjects [22,23]. 

The usefulness of a complete geriatric assessment was prospectively examined in 2000 Belgian patients [24]. This study established that multidimensional geriatric assessments gave prognostic information in terms of OS, morbidity and reduced QoL but, more notably, detected multiple difficulties that influenced the choice for a better targeted treatment in 30% of the patients. Recent randomized trials have now confirmed better treatment tolerability and improved QoL when CGA is combined with multimodal interventions for the various frailty issues detected in the assessment [25,26]. 

The main limitation of CGA is the time required for the evaluation and simple tools are eagerly awaited. In diffuse large B-cell lymphoma (DLBCL) elderly patients, an Italian group has developed a scoring system defining three groups based on age, ADL, IADL and the Cumulative Illness Rating Scale (CIRS). This tool permitted to identify a cohort of frail patients who should receive palliative treatment, and two other groups for whom a multidisciplinary discussion should establish the optimal treatment that can vary from a single pill to immune-chemotherapy [27,28]. 

#### 1.2.2. Disease-Related Issues

G8 screening scale is a simple tool for fitness assessment that has been validated in solid tumors and malignant hemopathies [29]. However, in “clinically fit” patients, the impact of nutritional and psychological status—in the G8 screening—is too important. Indeed, many DLBCL patients experience weight loss and often feel tired at diagnosis but their G8 score improves after one week of steroids, due to the reversibility of these lymphoma-related comorbidities. The strict application of the G8 screening added to a CGA could lead to an overestimation of frailty and the under-treatment of patients, undermining their chances to be cured [30]. Comorbidities are known to increase treatment-related toxicities and it is common to reduce the doses of chemotherapy, reducing in some circumstances, the chance to be cured. In younger patients, a minor reduction in the dosage or dose intensity could reduce their chances to be cured [31,32].

At the time of diagnosis, it is thus critical to differentiate clinical manifestations due to the patient’s existing frailty or due to the disease itself. 

Older non-Hodgkin lymphoma (NHL) patients with advanced stage at diagnosis often present a more unfavorable biological profile [non-germinal center (GC) vs. GC], genetic mutations (MYC positive) and an increased incidence of anemia. Finally, lymphoma remains the major cause of death and should be treated adequately. 

Two groups (GELA and German) have both confirmed that R-CHOP21 is superior to chemotherapy alone [33]. The advantage of Rituximab was observed in all age groups, but this trial was restricted to “fit” patients below 80 years old. 

In a prospective phase II trial, N. Peyrade and colleagues examined the R-miniCHOP regimen in a group of 80+ years old DLBCL patients [34]. This trial established the practicability and effectiveness (49% OS at 4 years) of this attenuated R-CHOP in very old patients chosen on the basis of limited comorbidities. As already observed in retrospective studies, lower doses of adriamycin seem to be as effective as full doses for these patients [31]. Pharmacokinetic information is dramatically missing in this setting, but it is conceivable that older patients may have an augmented half-life of drugs or their metabolites. 

#### 1.2.3. Treatment-Related Issues

The major side effect of chemotherapy in older patients is neutropenia (up to 60% above 80 years) with a 9% to 23% mortality. Marrow reserves are weakened in patients above 60 years old but, fortunately, their response to growth-colony stimulating factor (G-CSF) is comparable to that of younger patients. For over 10 years, we have known that G-CSF as a primary prophylaxis decreases mortality in the treatment of NHL. However, in reality, only 36% of older patients receive G-CSF as a primary prophylaxis, and up to 30% receive no G-CSF at all [35].

In older patients with chemo-sensitive lymphoma, the first course of chemotherapy can lead to an early death due to the tumor lysis syndrome. The risk can be significantly controlled by a pre-phase treatment of steroids with or without Rituximab [36]. 

Other concerns after treatment with R-CHOP are their sensitivity to cardio-vascular problems and late heart failure, but also to diabetes (steroids) and polyneuropathies (vincristine). Particularly, neurotoxicity can lead to functional and cognitive decline, resulting in a loss of autonomy. This regimen also increases the risk of secondary cancers such as lung cancers and myelodysplastic syndrome (MDS).

## 2. How to Prevent Toxicity 

In this section, we will discuss the most common treatment-related complications that can benefit from supportive care.

### 2.1. Prevention of Neutropenia 

Neutropenia increases the risk of life-threatening infections, often leading to the loss of dose density/intensity and the worst disease outcome [37].

Neutropenia is higher in the first cycle of treatment [38]. These observations support the use of G-CSF in “primary” prophylaxis for older patients [39,40]. G-CSF is a protein which stimulates the maturation, survival and differentiation of bone marrow myeloid progenitors [41]. The recombinant form of G-CSF, filgrastim, is also associated with a 40% reduced risk of febrile neutropenia (FN) and increased survival [42]. A biosimilar of G-CSF provides wider access and this lower incidence of FN translates into an economic benefit [43,44]. 

Several trials and meta-analyses have demonstrated that G-CSF as a primary prophylaxis decreases the duration and severity of neutropenia and reduces the incidence of FN [45,46]. Primary prophylaxis is thus strongly recommended for regimens with a risk of FN ≥ 20% [47,48,49]. However, age above 65 years is considered an independent risk factor and also represents an indication for filgrastim prophylaxis for protocols associated with only 10–20% of risk. An advanced stage disease, prior FN, the absence of antibiotic prophylaxis, low performance status, a poor nutritional score, cardiovascular frailty and kidney and liver insufficiencies have also been identified as potential risk factors [50].

Long-acting G-CSF with a bigger molecular size can avoid renal clearance and are cleared mainly by circulating neutrophils. This explains an increased clearance when neutrophils recover [51,52].

Multiple trials have demonstrated that peg-filgrastim prophylaxis compared to short-acting G-CSF is associated with a lower incidence of FN and related hospitalization.

This may be explained by a lack of compliance (daily injection for 7–10 days with G-CSF versus one administration per cycle for peg-filgrastim), while others suggest that the superior efficacy of the long-acting G-CSF is related to the clearance mechanism by neutrophils, causing a well-adjusted efficacy in neutropenic patients. Peg-filgrastim is now favored over the short-acting G-CSF [51,52]. 

### 2.2. Lymphopenia and Infections: Pneumocystis jirovecii and Antiviral Prophylaxis

Lymphopenia is commonly found during treatment for lymphoproliferative disorders and it is well known to be associated with an increased risk of infection and infection-related death [53]. *Pneumocystis jirovecii* (PJ) is a ubiquitous microorganism first described as a cause of interstitial pneumonia in immunocompromised children [54]. Later on, it became a major pathogen in patients receiving immunosuppressant drugs, steroids or chemotherapy. The mortality rate of PJ pneumonia varies between 30% and 60% despite adequate treatment, probably due to be the delay in diagnosis.

It is known that a CD4+ lymphocyte count below 200 cells/mm^3^ is a major risk factor for PJ pneumonia, but several other cases were published with a normal CD4+ lymphocyte count [55,56,57]. Initiation of PJ prophylaxis is thus recommended for patients associated with a >3.5% risk for PJ pneumonia. These are patients who receive severe lymphodepleting therapies such as prednisolone 20 mg/d or more for at least four weeks, or rituximab, bendamustine or kinase inhibitors [47,49,58,59]. Duration of PJ prophylaxis is recommended until normalization of CD4+ counts [59].

Refs. [60,61,62] suggest trimethoprim/sulfamethoxazole (TMP/SMX) is the first choice prophylaxis and has dramatically reduced the incidence of PJ infection [63]. 

Data concerning the association between rituximab and increased viral infections are poor; however, several cases of reactivation of chronic HBV infection and herpes zoster have been described [64]. It is mandatory to screen lymphoma patients for HBV serology and administer antiviral prophylaxis in case of HBsAg positivity; if possible, entecavir or tenofovir must be preferred over lamivudine (less risk of resistance), and treatment should not be interrupted until 12–18 months after the end of anti-lymphoma therapy [65,66]. The positive impact of acyclovir on the reduction of the incidence of herpetic infections during chemotherapy has been known for four decades and is nowadays widely proposed in most regimens used for the treatment of lymphoproliferative disorders [67]. Recently, the recombinant zoster vaccine Shingrix showed important antiviral activity which was confirmed also in very elderly (>80 years) patients, particularly when treated with the two-dose scheme [68].

### 2.3. Monitoring of Thrombocytopenia

Thrombocytopenia is frequently observed during chemotherapy for hematological malignancies, ranging from 40% to 68%, and is often associated with anemia [51,69]. It has been widely described that platelet count decreases with age, with a more significant reduction observed in male patients [70,71]. Currently, there is no preventive treatment for thrombocytopenia, which is a frequent cause of loss of dose intensity. Several clinical trials are evaluating the potential benefit of using thrombopoietin receptor agonists (TPO-RA) such as romiplostim, eltrombopag, avatrombopag, lusutrombopag and hetrombopag, for the treatment of chemotherapy-related thrombocytopenia. Particularly, the use of TPO-RA such as romiplostim and eltrombopag in treating hematological malignancies such as lymphoma and myeloma has been associated with increased dose intensity, reduced bleeding and the need for platelets transfusion [52,72]. Despite no trial directly comparing the efficacy of these drugs to the patients’ age, it is reasonable to believe that the older population could benefit the most from a wider utilization. However, nowadays, TPO-RA are not approved for this indication and their employment is still limited to clinical trials.

### 2.4. Control of Anemia and Fatigue 

Anemia is commonly reported at diagnosis in older onco-hematological patients due to the numerous predisposing factors, such as reduced marrow reserve, comorbidities, polymedication, chronic inflammation, iron or other vitamin deficiency, malnutrition and hormonal abnormalities [73,74]. Furthermore, in older patients, the erythropoietin feedback loop seems to be impaired, especially for those presenting high levels of circulating pro-inflammatory cytokines, with erythrocytes losing sensitivity to erythropoietin stimulation [75]. For all these reasons, anemia in very elderly patients (>80 years) is highly prevalent; almost 25% for non-hospitalized subjects and up to 40% for in-patients [76]. 

Anemia in older patients is also associated with fatigue, loss of autonomy, functional dependence and limited social activities [77]. Cancer and its treatments, both chemo- and radiotherapy, are known to reduce hematopoietic activity and increase chronic inflammation. The incidence and the severity of anemia increases after each additional cycle of antineoplastic treatment [78]. Cancer-associated anemia can be treated with erythropoiesis-stimulating agents (ESA), such as epoetin and darbepoetin alfa, to reduce the burden of anemia-related symptoms and the need for a transfusion [70]. The efficacy of these agents is not immediate, but takes 7–14 days to occur. Efficacy data showed a similar activity among young and older patients in terms of hemoglobin increase and a lower need for transfusion, although a QoL improvement was reported mainly by younger subjects [50]. Long-acting formulation is now available, presenting similar efficacy and economic outcomes compared to short-acting ESA [79]. 

Some randomized trials raised concern about the correlation between ESA and thromboembolism and reduced survival, especially when using a hemoglobin target of 13 g/dL; this concern is still a matter of debate, since other meta-analysis did not confirm these data [80]. For this reason, ESA is approved only in the context of palliative treatments and must be discontinued after reaching hemoglobin levels of 12 g/dL. According to NCCN guidelines, anemia occurring during chemotherapy given with curative intent must be managed with red blood cell (RBC) transfusions [81]. 

### 2.5. Screening for Hypogammaglobulinemia (IgG < 4.5 g/L) and Infections 

Secondary hypogammaglobulinemia is commonly found in hematological patients and it is mostly related to anti-neoplastic treatments. Expert consensus identified severe hypogammaglobulinemia for IgG concentration of <4 g/L and mild hypogammaglobulinemia for levels ranging from 4 to 6 g/L [82]. However, the depth of hypogammaglobulinemia does not always correlate to an increased frequency of infections. Many patients that have almost normal levels of IgG present recurrent and severe infections [83]. There are other risk factors that have been associated with hypogammaglobulinemia and increased risk of infections: age, protein deficiency, malnutrition and trauma [84]. In the case of hematological cancers, hypogammaglobulinemia is well known for being associated with chronic lymphocytic leukemia (CLL), multiple myeloma (MM) and NHL [85]. The prevalence of hypogammaglobulinemia at diagnosis is 85% in CLL, 50–90% in MM and 15% in NHL [86,87,88]. In these patients, the efficacy of vaccination may be limited while prophylactic antibiotics administration has been linked to controversial results, raising concerns about the build-up of resistance [84,89]. Immunoglobulins replacement therapy represents, today, the best option for reducing the risk of infectious complications in patients presenting hypogammaglobulinemia [90]. Usually, the indication to start the treatment using 0.4 g/kg intravenous immunoglobulins (IVIG) relies on the presence of multiple bacterial infections combined with severe hypogammaglobulinemia (<4.5 g/L) [82,84].

### 2.6. Pre-Phase Treatment to Reduce Early Death and Tumor Lysis Syndrome 

Tumor lysis syndrome (TLS) is a life-threatening emergency but is easily preventable. In older patients with DLBCL, the first course of chemotherapy is responsible for the high mortality rate and a pre-phase treatment using steroids prevents the risk of TLS.

TLS is due to the fast lysis of malignant cells determining hyperuricemia, hyperkaliemia, hypocalcemia and hyperphosphatemia. Uric acid and calcium phosphate crystals in the renal tubules may lead to acute renal failure. 

The Cairo–Bishop definition identifies a laboratory and a clinical TLS [91]. 

Laboratory TLS is defined as two or more of the following metabolic abnormalities: hyperuricemia, hyperkaliemia, hyperphosphatemia or hypocalcemia. 

Clinical TLS is defined as laboratory TLS with one or more of the following clinical abnormalities: elevated creatinine levels (>50% above baseline), cardiac arrhythmia, sudden death or seizures. The diagnosis of clinical TLS is further categorized by the Cairo–Bishop grading classification from grade I (mild) to grade V (life-threatening). 

The optimal approach is thus prophylactic, using intravenous hydration and the use of urate-lowering therapy such as allopurinol, a xanthine oxidase inhibitor or Rasburicase, a recombinant urate oxidase that catalyzes uric acid into allantoin, which is ten times more soluble than uric acid. Rasburicase is preferred in high-risk patients because it acutely decreases the levels of uric acid; however, it should not be used with 6-glucosis phosphate deficiency because of the risk of severe hemolysis. 

Patients with Burkitt lymphoma (BL) are considered at very high risk of TLS, whereas patients with DLBCL, T-cell lymphoma and blastoid mantle cell lymphoma (MCL) are considered as intermediate/high risk, according to the presence of bulky disease or increased serum LDH levels.

### 2.7. Screening for Impaired Cognition-Evaluation of Depression 

“Mild” cognitive impairment is usually poorly investigated and thus underestimated in older cancer patients. In a population of “clinically fit “lymphoma patients who were prescribed chemotherapy, we reported that 30% of the patients presented an abnormal MMSE and 51% had an abnormal (<26) Montreal Cognitive Assessment (MOCA), a more sensitive test for executive memory. This cognitive impairment could reach 70% in patients over 80 years. This translated into a significant impact on 2-year OS rate (*p* < 0.01) [92,93]. 

In addition to cognitive impairment, we analyzed in our population three independent prognostic factors: low serum albumin, increased C-reactive protein (CRP) and patient’s comorbidities (two or more). We observed that the presence of these four factors was associated with a median OS below 6 months, significantly different (*p* < 0.001) when compared to age-matched patients with none of these prognostic factors. This score was called HEMA4 and is currently investigated in other centers for validation and also for better intervention when cognitive frailty is detected [20].

### 2.8. Screening for Cardiovascular Vulnerabilities 

Many cardiovascular side effects including QT prolongation, cardiomyopathy, myocardial infarction and pulmonary hypertension are attributed to radio-chemotherapy [94,95].

The usual side effects of anthracyclines are left ventricular dysfunction and heart failure, followed by arrhythmias and myocarditis [96]. It is generally agreed that a cumulative dose higher than 450–500 mg/m² of doxorubicin increases heart failure [97]. This threshold should, thus, not be crossed. Other risk factors of anthracycline are a prior history of heart failure and age > 65 years.

The definition of chemotherapy-related cardiotoxicity in the European Society of Cardiology (ESC) guidelines is a 10+% decrease in the left ventricular ejection fraction. Troponin level and brain-type natriuretic peptide (BNP) are currently investigated as other predictive markers of cardiotoxicity [39,98]. 

All preventive treatments with ACE-inhibitors, beta-blockers and angiotensin II receptor blockers have been disappointing in practice.

Anthracyclines are key drugs in the treatment of DLBCL. The GELA trial, with a 50% reduced dose of anthracycline, leads to similar complete remission and OS rates, suggesting that older patients may have an increased half-life of drugs or drug metabolites [34]. Another group published comparable observations in a retrospective analysis of older (80+) DLBCL patients where < 85% anthracycline dose intensity improved OS [99].

### 2.9. Restraints on the Use of Steroids 

Patients with DLBCL have to be treated during long periods of immobility, commonly due to treatment-related fatigue, which is known to reduce their physical performance. Functional performance decline is often associated with the cancer itself; however, cancer treatment and steroids, commonly used in lymphoma, are responsible for rapid proximal amyotrophy in older patients. This situation increases the risk of falls and requires a rational use of steroids. Older patients are also more sensitive to secondary diabetes, so glucose level monitoring is mandatory. 

Physical exercise added to standard care is now recommended to reduce fatigue and even depression. The inability to perform activities in daily life diminishes the QoL and can influence the choice of therapy [100].

The summary of preventive treatments for elderly NHL patients is shown in Table 1.

## 3. Management of Side Effects and Communication Improve QoL

For each patient, it is important to determine the risk/benefit ratio. The treatment paradigm in older patients with aggressive NHL is the choice between a conservative treatment that preserves QoL and an intensive and potentially curative treatment associated with a higher risk of toxicities. The patient should be involved in the discussion, be well informed and able to express his wishes regarding his QoL. 

QoL is an important outcome in the treatment of older patients with cancer. We have contributed to a prospective multicenter trial investigating QoL before and during treatment in order to determine predictive factors for QoL decline. In this cohort, all patients (70+ years) underwent CGA and baseline QoL evaluation with a follow up at 3 months. QoL decline was defined as a difference of at least 10 points compared to the baseline score. 

In a recent article, baseline QoL was significantly correlated to performance status (0/1 vs. ≥2), age, impaired IADL, pain, fatigue, reduced cognitive capacities and nutritional status. During the treatment, a decline in QoL was observed in 28% of patients and correlated with the stage of the disease, baseline pain, fatigue and malnutrition. However, direct interventions against these factors can subsequently improve QoL in these patients [8]. 

Finally, as accurately pointed out by M. E. Hamaker, most of the ongoing studies in elderly patients with malignant hemopathies do not address the correct endpoints [29]. Actually, the most important concerns in this population, such as QoL, health-care utilization and loss of functional capacity, were investigated in less than 10% of the trials. Future clinical randomized trials in old and very old (80+ years) patients should include these important additional questions.

## 4. Conclusions

Lymphomas are diseases frequently seen in the elderly, representing a therapeutic challenge in this setting due to the extensive use of chemo-immunotherapies which can be associated with a high burden of side effects and loss of autonomy. 

Nowadays, it is crucial to optimize the identification of the right candidates for curative treatments and to increase the tolerance to chemotherapy regimens for these patients through an extensive application of support therapy, not only to provide a high cure rate but also a good QoL. 

## Figures and Tables

**Table 1:** Prevention of major side effects in older patients with non-Hodgkin lymphoma.

**Neutropenia** (>60% above 80y, 40% grade III) and febrile neutropenia (mortality rate: 9–23%)	➢ **G-CSF/Peg G-CSF** **IVIG, TMP/SMX** …if required
**Anemia** (with low EPO response)	➢ Erythropoiesis stimulating agents
**Cardiovascular** problems (arterial hypertension, atrial fibrillation, late heart failure)	➢ **Cardiovascular** monitoring
**Tumor lysis syndrome** (most important toxicity and toxic death during first cycle)	➢ **Rasburicase** or allopurinol administration **Prephase** with Steroids
**Diabetes**	➢ Cautious administration of **steroids and vincristine**
**Drugs Interactions**	➢ Be attentive of **polypharmacy**
**Neuropathies**	➢ Reduced doses of **vincristine**
**Secondary tumor** (late event: lung, MDS)	➢ **Long-term follow-up** for second tumors

EPO = erythropoietin, G-CSF = growth-colony stimulating factor, IVIG = intravenous immunoglobulins, TMP/SMX = trimethoprim/sulfamethoxazole, MDS = myelodysplastic syndrome.
